# Controller–Pilot Data Link Communication Security

**DOI:** 10.3390/s18051636

**Published:** 2018-05-20

**Authors:** Andrei Gurtov, Tatiana Polishchuk, Max Wernberg

**Affiliations:** 1 Department of Computer and Information Science, Linköping University, Campus Valla, SE-581 83 Linköping, Sweden; 2 Communications and Transport Systems, Linköping University, Campus Norrköping, SE-601 74 Norrköping, Sweden, tatiana.polishchuk@liu.se (T.P.); maxwe333@student.liu.se (M.W.)

**Keywords:** cybersecurity, air traffic communication, controller–pilot data link, privacy, threat model

## Abstract

The increased utilization of the new types of cockpit communications, including controller–pilot data link communications (CPDLC), puts the airplane at higher risk of hacking or interference than ever before. We review the technological characteristics and properties of the CPDLC and construct the corresponding threat model. Based on the limitations imposed by the system parameters, we propose several solutions for the improved security of the data messaging communication used in air traffic management (ATM). We discuss the applicability of elliptical curve cryptography (ECC), protected aircraft communications addressing and reporting systems (PACARs) and the Host Identity Protocol (HIP) as possible countermeasures to the identified security threats. In addition, we consider identity-defined networking (IDN) as an example of a genuine security solution which implies global changes in the whole air traffic communication system.

## 1. Introduction

The aeronautical telecommunications network is utilized to provide air traffic communication (ATC) within the domestic airspace. The standard method of communication between an air traffic controller and a pilot is voice radio, using either very high frequency (VHF) bands for line-of-sight communication or High Frequency (HF) bands for long-distance communication. Controller–pilot data link communications is a two-way data-link system by which controllers can transmit strategic messages to an aircraft as an alternative to voice communications. CPDLC enables controllers to issue ATC clearances (level assignments, lateral deviations/vectoring, speed assignments, etc.), radio frequency assignments, and various free text requests for information. It also plays an instrumental role in reducing mid-air collision risk, while also decreasing voice traffic on radio frequencies. Voice communications provide a situational awareness that the data link does not, since unlike the voice communications, CPDLC clearances are intended to be seen by only the controller and the pilot.

Some studies [1] have discovered there is an urgent need to protect air traffic management (ATM)-related technologies from a wide spectrum of cyberattacks, and the solutions need to be implemented in a timely manner. With the widespread availability of cheap and powerful tools such as software-defined radios (SDRs), the aviation community has lost the considerable technical advantage that protected its communication over the past decades. Anyone possessing relatively cheap radio equipment (e.g., SDRs) can access and monitor data link communications and easily decode these messages. As outlined in [2], hobbyists create websites to share what they are hearing and distribute the data they collect via websites and social networks, creating a complete operating picture including aircraft ownership, aircraft type, flight plan, flight destination, and private information.

Aircraft communications addressing and reporting systems (ACARS) in general [3], and CPDLC in particular, have become an easy target for cybersecurity attacks. According to the Civil Air Navigation Service Organization (CANSO) 2020 security strategy [4]: “The introduction of increased automation and reliance on system-wide exchange of data means that the associated cybersecurity issues are now a key area that requires joint industry solutions”.

In this paper we review the technological characteristics and parameters of the CPDLC technology, based on which we construct a threat model and propose a set of possible countermeasures. We emphasize the importance of creating cybersecurity threat model which identifies all possible cyberthreats to ATM information systems, enabling the ATC authorities to be proactive and comprehensive in their approach to cybersecurity. We propose several solutions for the improved CPDLC security such as elliptical curve cryptography (ECC), protected aircraft communications addressing and reporting systems (PACARs) and the Host Identify Protocol (HIP), and discuss their performance and deployability in the view of the current state of the data messaging system. In addition, we consider identity-defined networking ( IDN) as an example genuine security solution, and conclude that implementing such a standard within the whole communication system at the scope of the air traffic control unit or broader implies global system changes and may require several years of standardization and adaptation, and significant resource consumption.

The rest of the article is organized as follows. In Section 2 we analyze the related work. We review the technical characteristics of CPDLC and the main principles of this communication technology in Section 3. In Section 4 we present the threat model and analyze possible attacks on data link messaging system. We propose several solutions for CPDLC protection and consider an example genuine security proposal applicable to the whole aeronautical system in Section 5. Section 6 concludes the paper and outlines the future work directions.

## 2. Related Work

The research community is questioning the security of many of the aviation systems [5]. In [6] Strohmeier et al. discuss the current lack of security within the automatic dependent surveillance – broadcast (ADS-B). In [1] Strohmeier mentions that CPDLC, along with other systems, is inherently insecure as it provides unauthenticated and unencrypted data links which are easy to eavesdrop upon and attack. In [7] the authors present an interesting experiment where a possible attack was simulated in a testing environment demonstrating how the CPDLC system can be attacked through a man-in-the-middle approach using freeware and open source tools. Although they did allow the attacker to have physical access to the network, which is unlikely to happen in real life, the work demonstrates that the system is insecure at the application level even before it goes into the wireless medium.

According to McParland et al. [8], air traffic navigation (ATN) systems should support authentication for peers, while securing the data integrity in order to ensure that the information has not been modified or duplicated. The questions are not new and several proposals on how to secure the ATN system were presented more than 15 years ago. In [8] the authors proposed how to secure the communication through encryption using an asymmetric key system to securely communicate with a symmetric session key. In addition, in the same year Olive [9] designed a complete model demonstrating how to secure the ATN system while minimizing security overhead. Some years later the authors of [10] suggested a secure protocol design based on elliptic curve cryptography (ECC). None of the proposed security solutions were adopted by the authorities, leaving CPDLC together with many other ATN means without any form of protection against cyberattacks.

## 3. Controller–Pilot Data Link Communication

The section gives an overview of controller–pilot data link communications (CPDLC). It describes the main principles, the different data link services, and the basics of CPDLC operation.

CPDLC is a wireless digital message-based communication system used in parts of today’s air traffic, enabling air traffic controllers and pilots to communicate via a data link. CPDLC is utilized as a secondary communication method, after the primary method of very high frequency radio (VHF-radio) [11]. VHF-radio is voice-based, half-duplex, and only works as fast as human speech allows. As the amount of air traffic steadily increases and is predicted to continue to increase in the future [12], voice communication systems become more and more congested. CPDLC provides the needed benefit of freeing up critical transmitting time for voice communication. Initial experiments demonstrated an about 84% reduction of voice space occupancy [13]. CPDLC reduces the risk of mishearing a clearance or other instructions, especially when a message contains multiple elements [1]. The workload of both air traffic controllers and pilots is reduced with the automation of event-driven reports and flight plan updating [14]. CPDLC greatly improves communication capabilities in oceanic areas, especially in situations where controllers and pilots have previously had to rely on a third-party HF communications relay. CPDLC uses the VHF Data Link Mode 2 (VDL2) with the frequency band from 118.000 to 136.975 MHz, and a data rate of 31.5 kilobits [15]. It connects through either the Aeronautical Telecommunications Network which covers mainly European, continental airspace, or the Future Air Navigation System FANS-1/A [16], a system that enables the possibility of connecting through a satellite communications network to provide the service in oceanic airspace.

**Figure 1:**
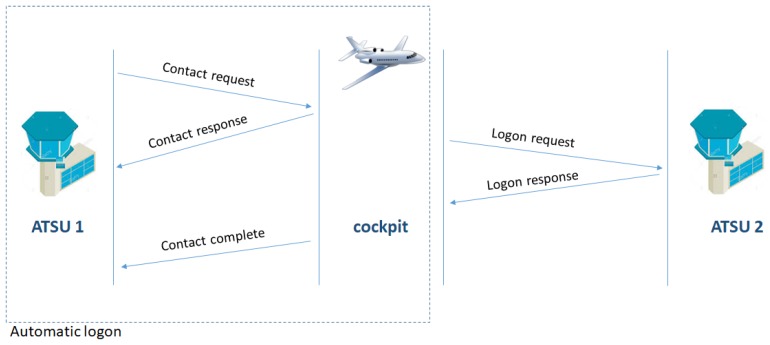
The controller–pilot data link communications (CPDLC) logon process. ATSU: Air Traffic Services Unit.

In order to connect and use CPDLC the aircraft crew has to perform a logon request. The logon is either an initial logon done by flight crew or a part of the forwarding procedure when the aircraft has to switch between two air traffic Air Traffic Services Units (ATSUs). An initial logon is done by the flight crew by entering the four-character identifier of the ATSU that the logon request is to be sent to. When the aircraft is to switch ATSU in flight, the system allows for a contact request to be sent, which triggers an automatic logon with the next ATSU, specified in the contact request message, initiated by the current ATSU (Figure 1).

**Figure 2:**
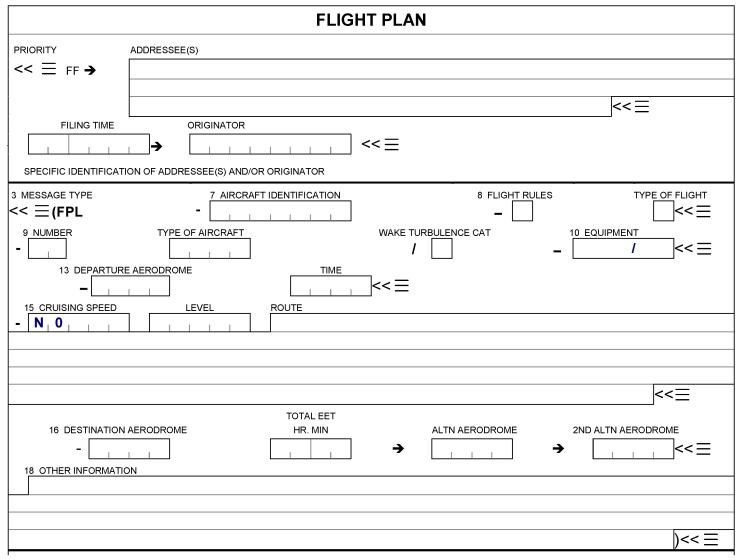
Example of a blank flight plan. (EET: estimated elapsed times.)

The logon request message contains the following information: aircraft identification (item 7 of the flight plan), aircraft registration and/or aircraft address (both in item 18 of the flight plan), and departure and destination aerodromes (items 13 and 16 of the flight plan). An example of a standard flight plan blanket is shown in Figure 2. The logon is done to provide the ATSU with information about what data link applications are supported by the aircraft system, identifying the aircraft and ensuring future messages will be delivered to the correct aircraft, and updates are made in the correct flight plan.

After the logon request has been successful, only then can a connection request be sent by the ATSU to the aircraft. The aircraft system will then, if no other connection exists, accept the connection request, establish an active connection, and send a connection confirmation message to the Current Data Authority (CDA). If the aircraft already has an active connection, and the aircraft system verifies the requesting ATSU as the Next Data Authority (NDA), it will instead accept the connection request and establish the connection as an inactive connection and send a confirmation reply to the CDA. Only the CDA can send a valid next data authority message which specifies the NDA by its four-character International Civil Aviation Organization (ICAO) identifier. In any other case the aircraft will reject the connection request and reply to the requesting ATSU with a rejection message.

Finally, the CDA sends a termination request which terminates the active connection and forces the aircraft system to establish an active connection with the next ATSU, converting the NDA to the new CDA.

## 4. Threat Model

We construct the threat model reflecting vulnerabilities of CPDLC technology. A cybersecurity threat model identifies all possible cyberthreats to ATM information systems, enabling the ATC authorities to be proactive and devote resources to addressing vulnerabilities ahead of time.

CPDLC messages are transferred over the data link, which does not provide any authentication or encryption. As a result, any outsider possessing radio equipment can monitor data link-based communications and decode these messages.

### 4.1. Possible Attacks against CPDLC Technology

CPDLC’s current lack of security puts it at risk of multiple possible threats, as summarized in Table 1.

**Eavesdropping** is performed when an unauthorized party listens to the data traffic without the permission of the communicating parties [17]. Eavesdropping is widely considered the easiest form of attack as it does not involve any active actions by the attacking party other than some necessary equipment to receive the signal, and the equipment needed to decrypt any encrypted information. In case of CPDLC this is made even easier as there is no current encryption in use. This attack does not directly influence any data, yet it is a straightforward way to map the usage of the CPDLC in the local area of the attack.

**Jamming** intends to deny the victim of the attack access to the service [18]. By jamming the receiver, the attacker intends to block the access to the service by reducing the channel’s capacity. This is done by filling the channel with enough noise to make it impossible to get any comprehensible data through to a receiving party. All users connected through a jammed node in a closed circuit network would be affected by a jamming attack, and the bottleneck connections become extra vulnerable to this kind of attack. In general, a wireless jamming attack is easy to detect using a directional receiver to probe for the direction of the jamming signal.

**Flooding** is very similar to jamming. Flooding aims to deny the victim’s access to the service. It is usually enforced by sending multiple packets of data to the same receiving party instead of filling the channel with noise. Incoming queries will start to queue up if the destination receives more queries than it can handle per given time frame. Other actions will likely be left unattended as the queue is preventing valid data from being processed in a timely manner. Ultimately, a system might time out under the stress and completely come to a standstill due to overload.

**Injection** is done by sending unauthorized messages. The information is said to be injected into the network by not originating from an authorized source. This type of attack could possibly be very severe and is difficult to detect if the system lacks necessary protection to check from what source the information is originated.

**Alteration** attack is defined when attacker alters the legitimate data. It manifests itself as data being modified, redirected to another recipient, or delayed. Despite the fact that the data is altered, it keeps its original validity which makes this type of attack hard to detect.

**Masquerading** means that the attacker impersonates an authorized user, be it an aircraft or ATC, and gains unauthorized privileges. Unless detected, the masquerading attacker has the means to commit to a full conversation with its victim. In case of CPDLC, an outsider may be capable of issuing instructions to the aircraft [17].

### 4.2. Threat Actors

According to [3], threat actors may be classified as passive or active.

An attacker is *passive* if he does not interfere with the medium. A passive attacker may have a moderate level of technical capability, i.e., can set up and use equipment such as an SDR and/or a V antenna. Given the range of uses for avionic data link, different attackers are thus likely to have various intentions. Primarily, an attacker is listening and collecting data to achieve competitive advantage or for some kind of surveillance. He does not harm the system directly, but the distribution of the specific knowledge about the aircraft may potentially be used by the active actors.

An *active* attacker interferes directly with the medium. He may be equipped with an SDR capable of transmission, amplifiers, transmit/receive (TX/RX) VHF antennas, and sensors in multiple locations, and may collect data in a mobile fashion. The corresponding equipment provides an active attacker with the capability of causing leaks of personal information, limited access to the avionic systems, message injection, modification, and deletion. Attackers seeking criminal gain might focus on financial or operational data, allowing them to steal assets or blackmail victims.

### 4.3. Requirements for a Secure Communication System

To provide the full protection against all possible attacks, the following general requirements for a secure communication system need to be fulfilled:

1. **Authentication**. According to [14], CPDLC has a very transparent authentication process. Attackers with malicious intent may easily fool it and a simple undetected mistake can lead an incorrect logon being processed, as it was described in [19]. In its current state, not the pilot but the aircraft computer authenticates a connection with the ATSU. The procedure may be changed so that every active pilot in the aircraft makes a personal authentication, thereby removing the dependency between authentication and the flight plan. A personalized authentication system will adjust to the pilots operating different aircraft at various times. Such a dynamic setup as in case of personalized pilot authentication might result in a complicated key management system.
2. **Confidentiality** of the CPDLC is not covered at all, as everything is sent in simple plain text without any encryption enforced. With the recent technological advancements enabling active attackers to perform intrusions into the system, it becomes possible to receive and decode the messages’ content and access the sensitive information, which can lead to a privacy breach for the participating parties.
3. **Integrity** is not guaranteed by the system either. Today’s use of the forward error correction protocol, a lightweight version of the Reed–Solomon code, is only meant to correct apparent errors upon receipt of the information being sent. Any information tampering through a man-in-the-middle attack might go through the system as a valid message. It is up to the human recipient of the information to question its validity, which is unlikely to occur if the information does not conflict with the original intentions of the aircraft/ATC. Integrity combined with authentication is a robust foundation to aim for when securing the data link communication. It should be able to assure that a message received is valid and trustworthy.
4. **Non-repudiation**. Traditionally, asymmetric key cryptography has been used to provide non-repudiation. A digital signature assures that the public key used for message encryption is associated with the secret key used to decrypt the information by a trustworthy party. It does not cover the cases where a secret key would be compromised.
5. **Availability**. The system currently lacks any means to protect itself against an attack aimed at disrupting its availability. The consequences of an attack may vary depending on whether a single aircraft is denied services or the ground station is targeted. An attack will also be restricted to a specific geographical area as long as the attacker lacks physical access to the whole aeronautical telecommunication network.

The response times of the system experiencing issues with its availability often increase above normal limits, which may lead to timeouts or complete denial of service. In this case, thoroughly developed backup procedures need to be specified in order to guarantee the robustness of the communication system and help to recover the affected network in a timely manner.

## 5. Potential Countermeasures

A critical factor defining the requirements for CPDLC system security is its limited available bandwidth. According to [20] CPDLC allows for up 31.5 kb/s and in practice the actual performance of the data link can drop down to only 4 kb/s. Any proposal for CPDLC protocol security has to take into account this limitation on the available bandwidth, and should avoid extra overheads.

In what follows we discuss several solutions to improve CPDLC security, taking into consideration the limitations on the system resources.

### 5.1. Protected Aircraft Communications Addressing and Reporting System (PACARS)

The commercial company Aeronautical Radio, Incorporated (ARINC) supplies a standard for what they call PACARS [2]. Originally developed for the military to protect their communication, ARINC claims to provide encrypted data link traffic for FANS 1/A. It supports data confidentiality, integrity, and authentication using ECC, which is a step in the correct direction towards securing the system. Currently it only supports FANS 1/A and is widely used [16], which does not necessarily guarantee that its implementation would universally secure the CPDLC system as it might not cover the European ATN.

PACARS is an application that can be added to the aircraft without hardware modifications and without burdening the system with message delays. Using the ARINC 823-defined compression, both message latency and overall message size are reduced. PACARS has been validated in several avionic manufacturers of data link Communication Management Units (CMUs). PACARS can also be added to other peripherals, such as the flight management systems (FMSs) and electronic flight bags (EFBs).

PACARS implementation seems to be costly and not well documented. We consider it promising in the view of its applicability to aircraft communications addressing and reporting systems (ACARSs) in general, and to CPDLC in particular, as a part of it. However, it is a closed commercial system, and future studies should aim at developing an open expandable architecture based on open standards.

### 5.2. Elliptical Curve Cryptography

Elliptical curve cryptography [10] uses relatively small keys while providing the same level of security as encryption techniques based on factoring of large prime numbers. As a result, less computational power is needed to encrypt and decrypt the information. According to [21], the computational overhead of ECC was significantly lower than the overhead associated with the widely used RSA (Rivest–Shamir–Adleman) cryptosystem. Table 2 presents the comparison for the message sizes encrypted with these two algorithms.

According to [22], a 161-bit ECC is roughly 5 to 10 times as fast as a 1024-bit RSA private key operation. Further comparisons show that as the key size increases so does the performance difference. Unfortunately, there is a problem with ECC certification. In 2015, the National Security Agency (NSA) announced its intentions to move to the new set of cryptographic algorithms to post-quantum cryptography (PQC). During the transition, the NSA advised the vendors, who decided to adopt the Suite B which includes ECC, to wait for the next upcoming set instead. The reason for this behavior has been a question within the cryptography community [23] which is still unanswered.

The latest SHA-3 hashing standard is based on a subset of the Keccak cryptographic family with sponge construction. It can be also extended to cover a stream cipher and authenticated encryption combining previously distinct functions in a single efficient implementation.

### 5.3. Host Identity Protocol (HIP)

The Host Identity Protocol (HIP) [24] is a new internetworking architecture developed at the Internet Engineering Task Force (IETF); it had its first stable version ready in 2007. HIP has been developed to be fully backwards compatible with today’s infrastructure of the Internet Protocol version 4 (IPv4) and the growing version 6 (IPv6) infrastructure to make it possible to implement in an already existing environment without changes needed to be done to either applications or the routing infrastructure of the network, and upgraded HIP hosts are still able to communicate with non-HIP hosts.

By implementing a new name space in the TCP/IP (Transmission Control Protocol/Internet Protocol) stack, a public key known as the Host Identifier, it disconnects the two roles of identifier and locator from each other in the IP address. The Host Identifier assumes the role of identifying the host and the IP address continues to work as a locator. This enables among other things a connection between two hosts to be kept open even if the two hosts are mobile and continuously change locations thereby enhancing connectivity and mobility [25].

Data packets will use the host identity as source address by having it converted into a Host Identity Tag (HIT), which looks like an IPv6 address using a predefined prefix, called Orchid. This HIT follows the packets through to the Internet Protocol Security (IPsec) Encapsulated PostScript (ESP) Security Association (SA), where it is yet again converted to an IP address and added to the header. This makes it possible for any non-HIP node in between the two communicating nodes to handle the packets like a normal IPsec ESP transport mode packet. When the packet is received at the other end it goes back through the IPsec module that, if it passes verification and integrity checks, discards the IP address and reapplies the HIT as the source address to the packet to assure upper layers of its validity. This ensures that the packet was created using the correct cryptographic protocol where the private key corresponding to the HIT was used and protects against IP spoofing.

HIP can approach unwanted traffic in two separate ways, either directly by hiding or in a way it makes the recipients directly inaccessible by forcing the sender to gain the consent of the recipient before a connection is established. The second way is to increase the cost of sending data, or inversely minimize it. This is accomplished by introducing a four-way handshake between the sender and recipient before any other data can be sent. Hiding the hosts is achieved by creating an overlaying system that takes care of the handshake between the sender and receiver, and only lets the sender get in direct connection with the recipient after this handshake has been accepted by the recipient and allowed a direct path through a HIP firewall.

The Host Identity Protocol has a potential to, if implemented for CPDLC protection, not only solve the first four requirements for the completely secure communication system, but also partially cover the availability through its way to handle unwanted traffic. It will function as a countermeasure to the two kinds of potential attacks: flooding and injection. A HIP-enabled host will discard any unauthorized data, but will not be protected against a jamming attack filling the medium with noise. Though the source of origin of a jamming attack is easily pinpointed using direction finding, it still poses a risk. The HIP appears to be a very effective in covering all aspects of secure communication. Concerning the HIP overhead, according to [24] ESP reduces the data rate by 32% in the wireless local area network (WLAN) access point (AP) network. More to the point, a Lightweight HIP (LHIP) [24] version has been developed to counter and reduce the impact of the increased overhead cost. By replacing the public-key cryptography, used for host authentication, in HIP with Interactive Hash Chain (IHC) authentication, the LHIP reduces the computational cost of HIP to less than 2.5% of the cost of a base exchange of normal HIP with 1024-bit RSA. Removing the use of an asymmetrical key system results in a lower level of security, which is resolved by signing distinct kinds of messages (update, close, and upgraded messages), with IHC signatures to reinforce the protection against man-in-the-middle attacks, as important control messages cannot be forged. Namespace security is an important part to prevent impersonation attacks and namespace conflicts. The LHIP is considered compatible as it does not change the way HIP works in general and has the ability to interact with HIP implementations. In addition, Diet HIP [26] is a protocol version that was specifically designed to function on resource-constrained devices and links, such as CPDLC.

### 5.4. Comparison of the Proposed Solutions

CPDLC technology was introduced without built-in security in mind. All three near-future security solutions presented above offer the possibility to be applied within the already existing system through modification.

The PACARS standard is based on ECC for both encryption and authorization. It lacks mechanisms to address system’s availability. Its documentation supports only FAN-1/A but lacks any information about the ATN-B1 used in continental Europe. We assume it is technically possible to apply it to ATN-B1 for encryption and authorization of ACARS in general and CPDLC in particular.

The HIP offers better opportunities to improve and fulfill all the five requirements of a secure communication. Not only does it incorporate the tools to encrypt the information, but also brings a secure authentication, including a foundation on how to handle keys using secure Domain Name System (DNS)-servers. However, it has been argued that the safekeeping of private keys will be a major issue during implementation of such systems. The reason to choose the HIP over PACARS is that this standard is supported and well documented, and shows a potential to be implemented in the future generation of digital datalinks within aviation, including new versions of CPDLC, whereas PACARS is limited to the current design of CPDLC and ACARS.

Further investigations should uncover the feasibility and applicability of the proposed measures in the narrowed scope for implementation.

### 5.5. Alternatives: Adopting Existing Security Standards

The Institute of Electrical and Electronics Engineers (IEEE) standardized protocols may be considered for adoption for the wireless communication links utilized in civil aviation. We saw such convergence in all domains, for example in cellular core networks where SS7 was replaced by TCP/IP. Internet of Things protocols such as IPv6 over the Low power Wireless Personal Area Network (6LoWPAN) or the Constrained Application Protocol (CoAP) might be adopted since those are used in lightweight sensor networks where low overhead and simplicity are critical factors. AeroMacs [27] should be mentioned as a successful step towards standardization. It is the only wireless technology that has been validated by the European Organization for the Safety of Air Navigation (EUROCONTROL), the Federal Aviation Administration (FAA), and the International Civil Aviation Organization (ICAO) to support the safety and regularity of flight. In future, we plan to evaluate applicability of the security proposals for lightweight networks with limited bandwidth described in [28,29] for securing air-to-ground communication channels currently utilized in aviation.

Achieving party authentication in aviation communication requires a source of trust. Cryptographic host identities for the HIP can be self-generated but require endorsement by a trusted third party. In Web security with the transport layer security (TLS) is traditionally implemented with certificates, which might be also applicable in the aviation sector. For instance, a country-wide aviation authority (e.g., Swedish ATNP Luftfartsverket (LFV)) may sign public keys associated with airplane or airport identity. If we assume that all airplanes and airports store all public keys of LFV and other nation’s authorities, it will be enough to authenticate airplane and airport communication. However, due to heavyweight nature of certificate, an alternative approach similar to the Domain Name System Security Extensions (DNSSEC) in the Internet could be used. A compact Host Identity Tag (HIT) representing a hash of the airplane or airport public key could be stored in public documentation and airplane flight plan. This does not even require changing the existing systems as short text fields are easily added there. It is sufficient to validate the authenticity of a public key during the key exchange. This approach may enable efficient authentication of parties during airplane-airport link establishment.

The IEEE recently published the new Recommended Practice Specification 802.15.9 for Key Management Protocols (KMP) for the constrained links [30], such as IEEE 802.15.4. Currently, the following KMPs are supported: HIP Base Exchange (HIP BEX), Diet HIP, 802.1X Internet standard, PANA (protocol for carrying authentication for network access), Internet Key Exchange version 2(IKEv2), and Dragonfly. A special message layer is defined that allows security encapsulation without presence of IP headers. It makes this specification highly relevant for securing constrained CPDLC.

### 5.6. Identity-Defined Networking

For the transport domain, security must be managed pro-actively over the system as a whole. This must also extend to include interfaces to critical supporting infrastructures such as communication networks and satellite systems. The challenge is to migrate these systems and infrastructures to a higher level of cybersecurity. Matt Shreeve, a technology policy expert from the aviation consultancy firm Helios, says that adding security systems retroactively can be an extremely costly and technically difficult process [31]. He advises building security in all new technologies and processes from the beginning.

The networking community has established a number of security standards and protocols, enabling the ATC authorities to adopt one or several of the existing standards for wireless technology and mobility. A representative example of generic security solutions for industrial communication networks was proposed by Tempered Networks [32].

Tempered Networks offer an identity-defined networking solution with military grade security that ‘cloaks’ critical infrastructure. They provide hardened, drop-in security appliances that enable airline data center technicians to easily create private overlay networks with unlimited scale. Airlines can easily segment and effectively hide their most critical networks and control systems that are utilized for in flight and ground operations.

The solution provides a secure overlay network among HIPswitch appliances that connects and secures an airline’s in flight or ground operations network and control systems. The HIPswitch Conductor orchestration engine coordinates configuration, security policies, trust relationships, monitoring, and analytics. The solution facilitates easy administration and provisioning of private overlay networks with delegated control, centralized governance, and oversight by IT. The approach provides the possibility to cloak the critical infrastructure, segment and extend the network, preserve legacy investment, easily add or revoke contractor/employee access, and troubleshoot and optimize your network.

The key capabilities of identity-defined networking are summarized below:

1. **Cloaking the Critical Infrastructure.** Devices can only be seen by trusted peers.
2. **Segmenting Networks.** Smaller, more manageable networks are more robust and secure.
3. **Increased Operational Integrity and Availability.** Visibility into network traffic enables diagnostics, debugging, and performance optimization.
4. **Secure Remote Access.** Highly constrained remote access that is simple to grant and revoke.
5. **Preserve Legacy Investments.** Integrates with your existing devices and infrastructure for in-depth security.

Implementing such a standard within the whole communication system at the scope of the air traffic control unit or broader implies global system changes and may require several years of standardization and adaptation, and significant resource consumption.

## 6. Conclusions

The importance of improving CPDLCs security and privacy stems from the need to create a secondary communication channel, trustworthy enough to alleviate an already congested communication VHF voice communication and enable ATC and its airspace capabilities for continued growth. Without an acceptable level of trustworthiness, CPDLC will work against its intended purpose by adding a greater workload in the already stressed environment of the air traffic control managing system.

We investigated CPDLC communication functions and its level of security and privacy in order to identify the risks and possible security threats. We have concluded that CPDLC communications lacks the appropriate level of cybersecurity. Improvements within a system are constrained by the aging structure of the foundation of the system, designed using legacy protocols. Implementation of any encryption methods therefore needs to have as small impact on the system’s performance as possible while still providing an all-round security protection.

A commercial solution, called Protected ACARS, claims to be able to solve the system’s lack of security using the ECC method. We argued towards a solution based on open IETF and IEEE standards. IETF protocols 6lowpan, COAP, and Diet HIP can be adopted for the design of the future aviation communication system. IEEE 802.15.9 best current practice also offers a set of Key Management Protocols for lightweight security in IPless CPDLC. We proposed utilizing the current flight plan and AIP information systems to provide a root of trust for authenticating CPDLC encryption.

Identity-defined networking was proposed as a generic solution to be applied to the air traffic communication system as a whole, including CPLDC and all the other air-to-ground and ground communication means utilized by air traffic control units. It can be incrementally deployed without the need to change the existing hardware.

In future studies, we plan to discuss the pros and cons of the suggested solutions with security experts within aviation, and proceed with testing and prototyping of the most promising ones to adjust them to the current needs of the Swedish and global aviation infrastructure.

## Figures and Tables

**Table 1:** Overview of possible types of attacks on CPDLC technology.

Threat Type	Actor Type	Affected Attributes	Example Attack
Eavesdropping	Passive	Confidentiality	Reading control messages
Jamming	Active	Availability	Channel blocking
Flooding	Active	Availability	Ground station/aircraft flooding
Injection	Active	Availability Confidentiality Integrity Non-repudiation	Ground station/aircraft ghost messaging
Alteration	Active	Integrity	Modification of message content
Masquerading	Active	Authentication Non-repudiation	Ghost aircraft/ground station identity

**Table 2:** Encrypted message sizes (in bits) for the RSA and elliptical curve cryptography (ECC) algorithms.

Encryption Algorithm	Public Key	Private Key	Encrypted Message
RSA	1088	2048	1024
ECC	161	160	321
